# Circulating serogroups of *Leptospira* in swine from a 7-year study in France (2011–2017)

**DOI:** 10.1186/s40813-022-00257-y

**Published:** 2022-04-04

**Authors:** Jeanne Naudet, Laurent Crespin, Julien Cappelle, Angeli Kodjo, Florence Ayral

**Affiliations:** 1 VetAgro Sup, Université de Lyon, USC 1233, Marcy L’Etoile, France; 2 Université Lyon, INRAE, VetAgro Sup, UMR EPIA, 69280 Marcy L’Etoile, France; 3 Université Clermont Auvergne, INRAE, VetAgro Sup, UMR EPIA, 63122 Saint-Genès- Champanelle, France; 4 UMR ASTRE, CIRAD, INRAE, Univ Montpellier, 34398 Montpellier, France; 5 Laboratoire des Leptospires, VetAgro Sup, Marcy L’Etoile, France

**Keywords:** *Leptospira*, Pig, Reproductive failure, Microagglutination test, Australis, Icterohaemorrhagiae

## Abstract

**Background:**

Leptospirosis is a widespread zoonotic disease caused by pathogenic *Leptospira* and is responsible for significant economic porcine livestock losses. Knowledge of *Leptospira* serogroups and their distributions is important for evaluation of the relevance of leptospirosis management measures, including use of the prophylactic vaccine that was recently made available in France. A retrospective study was conducted to determine the relationships between different circulating *Leptospira* serogroups. Pigs from across France presenting clinical signs suggestive of leptospirosis were tested with the microagglutination test (MAT) between 2011 and 2017. We used weighted averages to determine serogroup distributions according to MAT results and considering cross-reactions.

**Results:**

A total of 19,395 pig sera, mostly from Brittany, were tested, and 22.7% were found to be positive for at least one *Leptospira* serogroup. Analysis of the 4,346 seropositive results for which the putative infective serogroup could be defined, revealed that two out of ten serogroups were much more frequent than the others: Australis (48.5%) and Icterohaemorrhagiae (38.2%). Other serogroups, including Autumnalis, Panama, Ballum, Tarassovi, Sejroe, Grippotyphosa, Bataviae, and Pomona, were less common.

**Conclusions:**

Although diagnostic laboratory data cannot be extrapolated to infer the distribution of *Leptospira* serogroups at the nationwide scale in France, the analysis of such data can provide an overview of the relationship between circulating *Leptospira* serogroups in space and time. During the last decade, protection against the serogroups Australis and Icterohaemorrhagiae would have prevented most of the clinical porcine leptospirosis cases in the large number of farms that we studied. In the future, epidemiological information related to circulating *Leptospira* serogroups should be extracted from data with a standardized approach for use in nationwide or international surveillance and prophylactic strategy support.

## Introduction

Leptospirosis is a worldwide zoonotic disease of major importance caused by pathogenic spirochetes of the genus *Leptospira*. To date, 38 pathogenic *Leptospira* species have been described (comprising subclades 1 and 2, previously referred to as pathogenic and intermediary *Leptospira*, respectively) [1]. Among the currently known pathogenic *Leptospira* taxa, more than 300 serovars have been identified and classified into serogroups according to their antigenic similarities [2].

In both humans and other animals, *Leptospira* infection can cause mild or strong clinical signs or be asymptomatic. In swine, acute and chronic infections are described mainly with regard to reproductive impairments (abortion, stillbirth, and perinatal mortality) responsible for economic losses; however, deterioration of the general condition, including haemorrhage, haematuria, renal damage and death, has also been described [3]. Asymptomatic carriage also appears to occur in pigs, allowing for the undetected transmission and maintenance of the bacteria on farms [4].

Transmission generally occurs by contact with urine-contaminated water, in which the bacteria can survive for several months [5]. The main hosts of *Leptospira* bacteria are rodents; however, other wild and domestic mammal species can also be involved in the transmission pathways [6, 7]. Because pig farming is mainly conducted indoors in France, contamination is most likely via the introduction of an infected individual or through contact with commensal rodents. Furthermore, as close contact between animals can promote intraherd transmission of the bacteria, the regulation that has recently mandated that pregnant sows be grouped together (Directive 2008/120/EC) could lead to an increased number of *Leptospira-*infected sows.

Two stages of leptospirosis generally occur. The first is the acute phase, which occurs in the first weeks of infection, when the hosts may show clinical signs [7]. The second is the immune phase, which generally occurs in the second week of infection, when the host starts to produce antibodies against *Leptospira* [7]. During the acute phase, polymerase chain reaction (PCR) performed on blood is highly sensitive and can rapidly detect pathogenic *Leptospira* species [8–10]; nonetheless, when treatment is effective, negative results may occur. Culturing is less advantageous than PCR for early diagnosis; the culture method is time-consuming, and the isolation of *Leptospira* is rare. The microagglutination test (MAT) is a serologic method that detects only antibodies indicating a past or current infection [11]. Nonetheless, the MAT is the immunological reference standard for experimental leptospirosis diagnosis by the World Organization for Animal Health (OIE) and the World Health Organization (WHO) [12, 13]. Another supportive immunological test for the detection of antibodies is the enzyme-linked immunosorbent assay (ELISA) [14]. However, its diagnostic accuracy has not been completely established [8]. Among the *Leptospira* tests, the MAT has the advantage of a higher sensitivity and the potential identification of a particular serogroup using available reference strains. Cross-reactivity between serogroups frequently occurs in microagglutination testing and results from a lack of specificity, especially from predominant nonspecific IgM antibodies at the onset of infection [15]. In these cases, the MAT results involve titres directed against two or more serogroups, thus preventing determination of the infecting serogroup.

The burden of *Leptospira* on pig farms in Europe remains unknown. However, epidemiological information has been extracted from laboratory MAT data for diagnostic purposes. According to these data, between 19% and 26% of tested pigs were seropositive in Italy, Germany and France [16–19]. However, the inclusion criteria (e.g., the inclusion or exclusion of pigs without clinical suspicion of infection) and the serogroup panels varied among studies, limiting reliable comparisons of the results.

In addition, previous serological surveys do not report how the MAT results, including the cross sero-reactivity results, were managed [16–19]. According to previous studies, the interpretation of MAT results is subjective, and possible cross-reactions should be considered [20, 21]. Chappel et al. 2004 recommended that in the presence of cross-reactions, the serogroup associated with the maximum titre should be considered the predominant serogroup. In addition, Miller et al. (2011) showed that the values of a single dilution titre vary among operators. Thus, a strict difference of two dilutions between the maximum titre and the others should be considered to account for this variability and ensure that the putative infective serogroup is identified with sufficient evidence. As no consensus currently exists in the literature regarding the identification of the predominant serogroup, we suggest the use of weighted averages to evaluate the importance of circulating serogroups from large sets of MAT surveillance data. Weighted averages allow the assignment of higher weights to certain observations considered more reliable or important than others in the computation of the average [22].

Given that several mammalian species may act as reservoirs for swine leptospirosis, disease source control is complex. Vaccination may be a promising way to reduce the health and economic consequences of *Leptospira* infections. Vaccines provide protection against homologous or closely related but not heterologous serovars; therefore, particular attention should be given to identifying relevant serovar antigens for vaccine development. In France, a swine vaccine that protects against the following serogroups has been commercialized since 2019: Icterohaemorrhagiae, Australis, Grippotyphosa, Pomona and Tarassovi. However, a comprehensive survey of all serogroups circulating in swine herds in France is required to assess the relevance of the inclusion of a given serogroup in a putative vaccine. The last such survey dates back to 2007 [16], and in light of the spatiotemporal variation in serogroup distribution observed in domestic animals [23, 24], updated data are needed. Using a descriptive approach for the analysis of available laboratory data, our paper aims to report the results of circulating *Leptospira* serogroups from a large dataset obtained from swine herds in France.

## Materials and methods

For confirmatory diagnosis, 28,332 swine sera were collected by local veterinarians from pigs that showed clinical signs consistent with leptospirosis. The samples originated from throughout the country. The veterinarians requested the laboratory for MAT depending on their ability to recognize the clinical expression of the disease, which may vary among individuals, and consent of the owners. Thus, the collection of samples from farms with potential leptospirosis varied according to clinical signs, veterinarian experience and owner consent. In addition, because the sensitivity of the MAT may be low in the early infection stage or for specific serogroup infections [7, 25], samples from several pigs are recommended to increase the sensitivity of the diagnosis at farm level. However, no consensus regarding the number of individuals to be included for confirmatory analysis is available, which lead to variability in the number of contributions per farm. A farm was defined as the group of animals from which pig serum was sampled. It is thus an epidemiological unit. Altogether, the available data resulted from convenience sampling, allowing descriptive statistical analysis.

Serum samples were analysed by the Laboratoire des Leptospires (Marcy-l’Étoile, France) between October 2007 and April 2017 using the MAT to detect antibodies against a large panel of serogroups. Over the ten-year duration of the survey, the panel of serogroups varied slightly. However, 19,395 sera sampled between January 2011 and January 2017 were tested for the following serogroups, with their serovars in parentheses: Australis (munchen, australis, and bratislava), Autumnalis (autumnalis and bim), Ballum (ballum), Bataviae (bataviae), Icterohaemorrhagiae (icterohaemorrhagiae and copenhageni), Grippotyphosa (grippotyphosa and vanderhoedoni), Panama (panama and mangus), Pomona (pomona and mozdok), Sejroe (sejroe, saxkoebing, hardjo, and wolffi) and Tarassovi (tarassovi). This test was performed using a serum dilution series from 1:100 to 1:6400, and a titre $\ge$ 1:100 was considered suggestive of a past or current infection.

To avoid the limitations of cross-reactions and the subjective nature of MAT interpretation at the individual level [21], we used a weighted average approach. Weighted averages allowed us to assign higher weights to certain observations considered more reliable than others in the computation of the average [22]. Here, we assumed that a previously or currently infected individual would be associated with MAT seroreactivity directed against a single serogroup or a combination of two or three serogroups because of possible cross-reactions. Therefore, we used the number of serogroups detected in the combination of serogroups as the weight. These weights also ensured that each individual was given the same weight (1.00) in all computations.

More precisely, we applied the following criteria:

(1) A single predominant serogroup was defined by either a titre ≥ 1:100 against a single serogroup or a threefold or greater difference between the highest titre and the other titres. In both cases, a weight value of 1.00, associated with a high certainty of incrimination, was assigned to the predominant serogroup.

(2) Equally predominant serogroups were defined based on the detection of titres ≥ 1:100 against two or three serogroups with equal titres or a less than threefold difference between the highest titre and the next highest titre. The presence of two or three predominant serogroups most likely resulted from cross reactions [26]. Thus, weight values of 0.50 or 0.33 were allotted to the two or three predominant serogroups, respectively.

(3) Serological profiles with more than three predominant serogroups were considered uninformative and were removed from the data analysis, as these profiles likely resulted from cross-reactive IgM antibodies at the onset of infection [15, 21, 27].

The frequency of each serogroup was computed as a weighted average by summing the numbers of positive swine sera per serogroup, weighting by the values defined above, and dividing by the total number of positive sera [22].

A seropositive farm was a farm in which at least one individual tested positive. Each seropositive farm was associated with one or more serogroups depending on the MAT profile obtained from each tested pig. The serogroups computed at the farm level included all seroreactive serogroups regardless of the number of pigs reactive against each serogroup. For instance, a farm with two pigs that were seroreactive against the serogroup Australis would be associated with that serogroup. A farm with one pig seroreactive against the serogroup Australis and one pig seroreactive against the serogroups Icterohaemorrhagiae and Panama would be associated with the serogroups Australis, Icterohaemorrhagiae and Panama.

To describe the spatial serogroup distribution, mainland France was studied at the “département” scale (France is divided into 96 “départements”, administrative units similar to counties in the United States). The tested pigs in each “département” were visualized in Quantum GIS (QGIS version 2.18) with the background map from IGN GEOFLA®. Open access pig density data were used for pig distribution mapping [28].

To explore the temporal variability of the serogroups, the numbers of swine sera positive for a given serogroup weighted by the values defined above were retrieved for each year. These counts were analysed using generalized estimated equations (GEEs) for three reasons [29]. First, GEE models were developed from generalized linear models to accommodate correlated data [30]. In our study, the primary sampling unit was the farm and so some correlations among the counts of *Leptospira *circulating serogroups were expected at the farm level. Second, we were not interested in obtaining estimates for a particular farm but rather in describing the year-to-year variability among the relative numbers of serogroups at the scale of all farms (marginal models). Third, a limitation of GEE models is that they rely on the asymptotic normality of estimators for inferences [31]. With 1,114 farms sampled from 2011 to 2016 (2017 was discarded because the sampling was not complete for this year, with only ten farms sampled), we are confident that this approximation was reasonable for the data analysis. Because the data were count data, we used a log link function with a Poisson distribution. The model also included the number of seropositive individuals per farm as an offset variable. We followed the methods of Hardin & Hilbe [30], who recommended an unstructured working correlation matrix for complete datasets with few observations (three herein) by panels. PROC GENMOD was used to fit the GEE models (SAS Institute Inc. 2012. SAS/STAT Software, Version 9.4. Cary, NC).

## Results

**Fig. 1:**
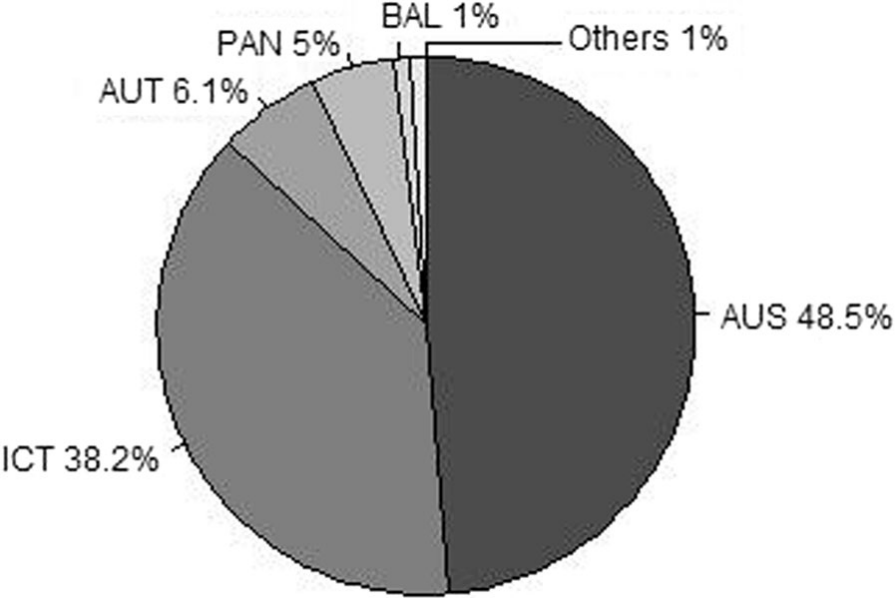
The distribution of *Leptospira* serogroups among 4,346 swine samples. Australis (AUS), Autumnalis (AUT), Ballum (BAL), Icterohaemorrhagiae (ICT), Panama (PAN)

Of the 19,395 tested swine sera, 4,398 (22.7%) were positive for at least one serogroup, including 2,608 that were positive for a single serogroup, 1,410 that were positive for two serogroups and 328 that were positive for three serogroups. Using the three criteria defined above, 52 MAT results (1.2% of the positive results) were seroreactive against more than three serogroups and were therefore excluded from the data analysis. Laboratory analysis using only the sera for which a single serogroup was unambiguously identified would have been based on 2,608 positive samples (59.3% of the samples testing positive), whereas our data analysis was based on 4,346 positive samples (98.8% of the samples testing positive), corresponding to a 66% increase in sample size. The predominant serogroups were Australis and Icterohaemorrhagiae, which had frequencies of 48.5% and 38.2%, respectively, followed by Autumnalis (6.1%), Panama (5%), Ballum (1.2%), Tarassovi (0.5%), Sejroe (0.2%), Grippotyphosa (0.2%), Bataviae (0.1%), and Pomona (< 0.1%) (Fig. 1).

**Fig. 2:**
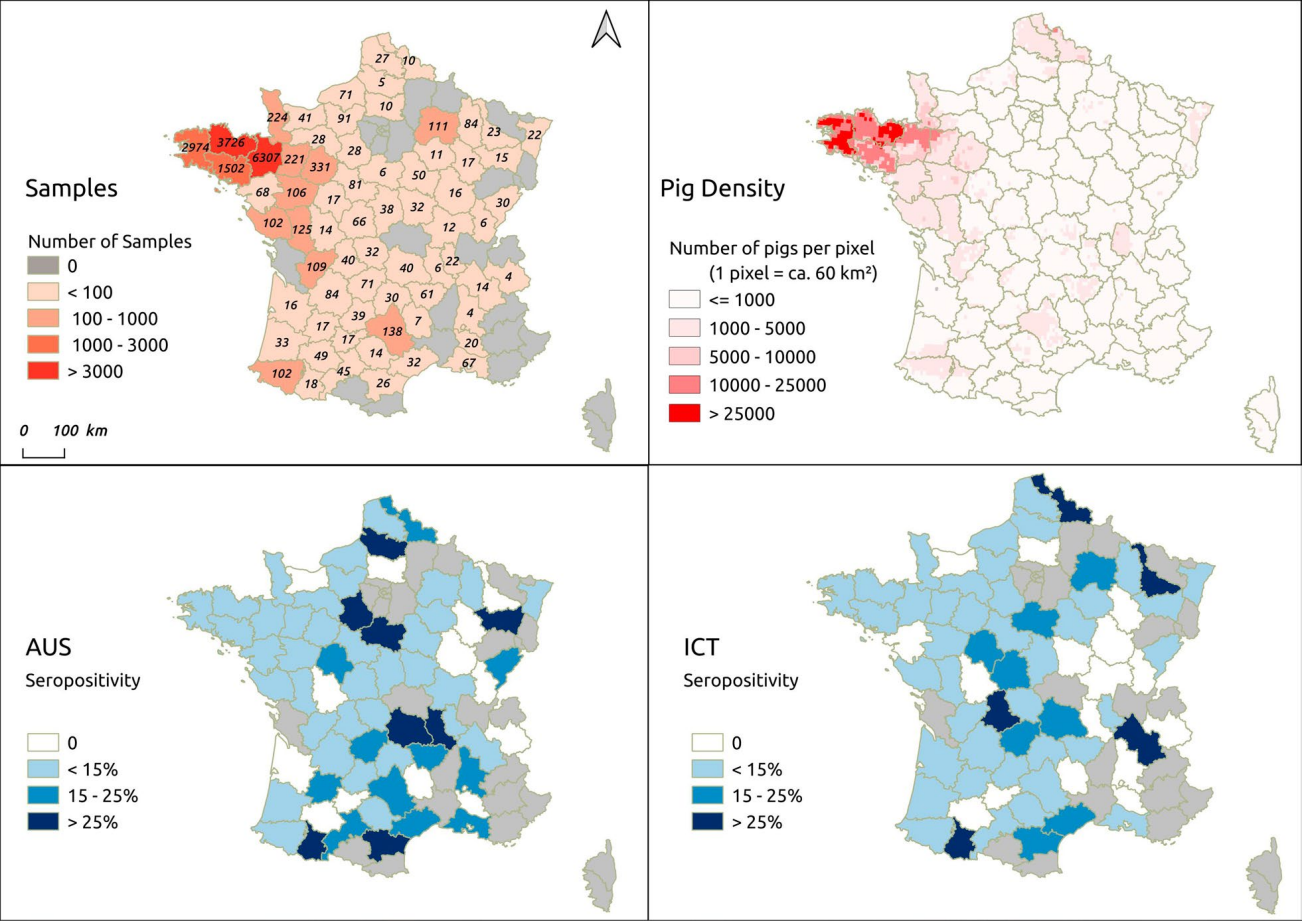
The spatial distribution of the swine tested and the seropositivity against Australis, Icterohaemorrhagiae per “département” (administrative district) in mainland France

The numbers of tested animals were higher in four “départements” of the Western France (n = 14,509, or 75% of the samples) than in those of the other regions. As shown in Fig. 2 (top right), Western France includes the vast majority of pigs reared in the country. Seropositivity against the predominant serogroups Australis and Icterohaemorrhagiae was retrieved in 55 and 52 of the 68 tested “départements”, respectively (Fig. 2).

Sera originated from a total of 2325 farms across France, among which 1124 (48%) had at least one seropositive individual. These farms were distributed in 87% (n = 59/68) of the tested “départements” in mainland France. The serogroups obtained at the farm level are shown in Table 1. More than 97.5% of the farms (n = 1,087) were associated with at least Australis and/or Icterohaemorrhagiae, and 50% (n = 561) were exclusively associated with Australis and/or Icterohaemorrhagiae.

**Table 1:** Number of farms having pigs with MAT profiles including Australis, Icterohaemorrhagiae and/or other serogroups

	*Leptospira* serogroups						
	Only Australis	Only Icterohaemorrhagiae	Only others	Australis and Icterohaemorrhagiae	Australis and others	Icterohaemorrhagiae and others	Australis, Icterohaemorrhagiae and others
Number of farms	134	94	27	333	66	51	409

The temporal distribution of the dataset, including seropositive vs. seronegative pigs and farms, is displayed in Table 2. Considering the temporal variability of the serogroups among years (Table 3), the fit of the model was adequate, as shown by the result that only seven Pearson residuals were larger than 3 (6 were less than 3.84 and one was 8.82, out of 3,342 observations) and that there was no overdispersion (normalized Pearson chi-square = 0.61). The relative counts of serogroups varied widely from year to year, as shown by the significant interactions (generalized score for serogroup×year: χ2 (DF = 10) = 203.79, p < 0.0001). The relative counts of Australis and Icterohaemorrhagiae fluctuated from year to year but nevertheless represented approximately 80% of infected individuals each year. The correlations among the serogroups were negative (Table, 4).

**Table 2:** Features of the dataset (n = 19,343) and distribution of the seropositive and seronegative pigs and farms over time from 2011 to 2017.

Year	2011	2012	2013	2014	2015	2016	2017
Total tested samples	**795**	**2770**	**3089**	**3965**	**3715**	**4846**	**163**
Number of seropositive pigs	190	550	597	988	664	1320	37
Number of seronegative pigs	605	2,220	2,492	2,977	3,051	3,526	126
Total tested farms	**100**	**333**	**347**	**497**	**459**	**568**	**21**
Number of tested positive farms	70	197	126	236	173	312	10
Number of tested negative farms	30	136	221	261	286	256	11
Minimum sample size per farm	1	1	1	1	1	1	1
Maximum sample size per farm	31	42	31	35	31	31	17
Median sample size per farm	7	7	8	7	7	8	8
Median number of seropositive samples per seropositive farm	2	2	4	4	3	4	3.5

Total tested pigs is the sum (in bold) of the two lines below (tested positive and negative). Total tested farms is the sum (in bold) of the two lines below (positive and negative)

**Table 3:** Weighted number of pigs with MAT profiles including Australis, Icterohaemorrhagiae and/or other serogroups between 2011 and 2017

*Leptospira* Serogroups	Years							Total
	2011	2012	2013	2014	2015	2016	2017	
Australis	86.67	224.17	242.50	343.50	376.17	802.33	24.00	2,099.33
Icterohaemorrhagiae	82.83	231.67	286.67	529.33	205.17	310.33	5.50	1,651.50
Others	20.50	94.17	67.83	115.17	82.67	207.33	7.50	595.17
Total	190	550	597	988	664	1320	37	4346

**Table 4:** Estimated correlations between serogroups from the unstructured working correlation matrix used with the generalized estimating equation model

	Australis	Icterohaemorrhagiae	Others
Australis	1.00	− 0.66	− 0.43
Icterohaemorrhagiae	− 0.66	1.00	− 0.26
Others	− 0.43	− 0.26	1.00

## Discussion

Employing a retrospective approach, our study shows that antibodies against pathogenic *Leptospira* serogroups were frequently detected on French pig farms. In addition, two serogroups were detected much more frequently than the others among seropositive pigs included, Australis and Icterohaemorrhagiae, with frequencies of 48.5% and 38.2%, respectively. These high frequencies suggested that among the 4,346 seropositive pigs, most associated *Leptospira* infections originated from only two serogroups out of the ten detected in this study.

The observed predominant seroreactivity against *Leptospira* serogroups Australis and Icterohaemorrhagiae in pigs was consistent with previous data reported in France [16]. This suggests the absence of any changes in the epidemiological context of *Leptospira* strain distribution. A recent study in Italy that included pigs with clinical suspicion of leptospirosis was implemented using an eight-serovar panel; seven serovars were the same as those in our panel, and their study identified Australis and Pomona as the most frequently detected serogroups [19]. However, caution is warranted in interpreting this consistency because the previous study do not report how the MAT results with cross-seroreactivity were managed, and the approach may have differed from that in the present study. These observations support the need for a standardized approach for MAT surveillance data analysis.

Previous studies have described a poor correlation between the presence of antibodies and the carrier state [25, 32]. Among 22 Australis-infected sows, six had titres above 1:100, which is the limit of positivity recommended by the OIE for screening and diagnosis [12, 25]. Our data underestimated the number of seroreactive samples against the serogroup Australis. However, we assume that the underestimation is the same among farms and over time, leading to a lower relative importance of the serogroup Australis in our results.

Regardless of the method used, the simple counts excluding any MAT result with cross reaction or the weighted average method, the results revealed predominance of the serogroups Australis and Icterohaemorrhagiae. The weighted average method allowed a consistent interpretation of the results among data and the inclusion of 66% more MAT results. The weighted average approach was thus useful for providing a more comprehensive overview of the results. In addition, it is an opportunity to standardize output of laboratory MAT results and to compare them among laboratories.

Australis and Icterohaemorrhagiae remained predominant in the farm-level analysis. This predominance was observed over time, even though the relative proportion of Australis and Icterohaemorrhagiae serogroups varied from one year to another. For anonymity reasons, it was not possible to identify farms that were potentially sampled multiple times during the study period; however, this number of farms was assumed negligible considering that our sampling was limited to 10% of the total pig farms in France (n = 22,000) [33]. In addition, the number of contributions (number of sampled pigs per farm) varied from one farm to another and may have had an impact on the results. Nonetheless, the contribution variation is unlikely to change the predominance of Australis and Icterohaemorrhagiae.

Regarding MAT, a previous experimental infection study showed that *Leptospira*-specific IgG (titres above 1:100) could be detected for more than 100 days [34]. Even when a threshold of positivity of 1:100 is applied, recently infected, chronically infected or previously exposed individuals cannot be clearly distinguished [4]. However, because we were chiefly interested in the distribution of the *Leptospira* serogroups at a large temporal scale, any evidence of past infection was relevant to the description of the serogroup distribution in pigs. In addition, the serogroups Australis and Icterohaemorrhagiae were predominant in each year from 2011 to 2016 in our dataset, which suggests that the misclassification of past or current infections would have limited effects on our results.

Most of the samples were obtained from Brittany, which is an intensive pig farming area compared with other regions in France. However, the serogroup distribution was similar among regions. The samples analysed originated from swine herds with clinical suspicion of leptospirosis; thus, we can conclude that throughout France, herds with reproductive disorders that were exposed to *Leptospira* were most likely exposed to the Australis and Icterohaemorrhagiae serogroups. Among the pig isolates related to the serogroup Australis, some were more likely to be associated with disease [35], and Australis exposure in pigs should be seriously considered as a potential cause of reproductive failure. A broader survey including nonsymptomatic herds could help clarify the relationship between *Leptospira* serogroup exposure and reproductive failure. However, given the low MAT titres found in infected sows and boars from farms where reproductive failure had occurred and where the serovar bratislava had been recovered from aborted sows, foetuses and boars, this may not be of value for the Australis serogroup.

Herd management of leptospirosis may rely on animal reservoir control. *Rattus norvegicus* is a frequent commensal rodent in livestock buildings and a selective carrier of *Leptospira* of the serogroup Icterohaemorrhagiae [36, 37]. Thus, rats may play a role to some extent in Icterohaemorrhagiae pig exposure, and this exposure could be reduced through rat management. As no selective carriers of *Leptospira* of the serogroup Australis have been described among commensal rodents, pigs themselves could be the main reservoir host in the context of pig farming, as suggested by Ellis [4]. Leptospirosis management through the culling of *Leptospira* carriers is limited by the low specificity and sensitivity of the MAT [38]. According to our results, which showed that more than 50% of the seropositive pigs were exposed to Australis and/or Icterohaemorrhagiae, management options preventing such infections could have greatly reduced the burden of the disease among the pigs.

As the serogroups Australis and Icterohaemorrhagiae are also pathogenic to humans, infected pig populations represent a potential cause of occupational disease, especially for breeders or slaughterhouse staff [39, 40]. Following a One Health approach, leptospirosis management in pig populations may contribute to the protection of human populations and to the mitigation of *Leptospira* persistence and transmission in the country.

## Conclusions

The analysis of data from diagnostic laboratories is useful for obtaining an indication of the circulating *Leptospira* serogroups in a region or a country and over time. Our results suggested that over the study period, most *Leptospira* infections in swine in France originated from only two serogroups, Australis and Icterohaemorrhagiae, out of the ten used for the laboratory analysis. This information should be considered to support future prophylactic measures.

In Europe, numerous laboratories have published interpreted MAT results. However, there is no consensus regarding serovars to be included in the panel, the positive threshold and serogroup determination in the case of cross-reactions, leading to the incomparability of results among studies. Therefore, this is an appeal to veterinary laboratories in Western Europe to standardize their approach to MAT surveillance data analysis. Based on our results and previously published results, future MAT surveillance data analysis in Western Europe should be supported by microagglutination testing with a common set of serovars belonging to the serogroups Australis, Ballum, Bataviae, Icterohaemorrhagiae, Grippotyphosa, Panama, Pomona, Sejroe and Tarassovi and using a weighted average approach to determine the serogroup distributions and considering cross-reactions.

## Data Availability

The datasets used and/or analysed during the current study are available from the corresponding author upon reasonable request.

## Abbreviations

MAT: Microagglutination test
AUS: Australis.
AUT: Autumnalis
BAT: Bataviae
GRI: Grippotyphosa
ICT: Icterohaemorrhagiae
PAN: Panama
POM: Pomona
PYR: Pyrogenes
TAR: Tarassovi
SJ: Sejroe
